# circ5912 suppresses cancer progression via inducing MET in bladder cancer

**DOI:** 10.18632/aging.102464

**Published:** 2019-12-05

**Authors:** Yinjie Su, Zehu Du, Guanglei Zhong, Yiyao Ya, Junming Bi, Juanyi Shi, Luping Chen, Wen Dong, Tianxin Lin

**Affiliations:** 1 The Department of Urology, Sun Yat-Sen Memorial Hospital, Sun Yat-Sen University, Guangzhou, China; 2 The Department of Thyroid Surgery, Sun Yat-Sen Memorial Hospital, Sun Yat-Sen University, Guangzhou, China; 3 The Department of Gynecological Oncology, Sun Yat-Sen Memorial Hospital, Sun Yat-Sen University, Guangzhou, China; 4 The Department of Urology, Guangzhou First People's Hospital, School of Medicine, South China University of Technology, Guangzhou, China; 5 The Department of Pediatric Surgery, Sun Yat-Sen Memorial Hospital, Sun Yat-Sen University, Guangzhou, China

**Keywords:** bladder cancer, circ5912, MET

## Abstract

Background: Increasing evidence suggests that circular RNAs play a key role in regulating bladder cancer progression. However, this remains to be fully elucidated.

Results: In this study, we reanalyzed our previous RNA sequence, and circ5912 was found to downregulate significantly in bladder cancer tissues compared with normal control. Expression of circ5912 inversely correlates with bladder cancer grade, stage, metastasis, and better patient outcomes. *In vitro* and *in vivo*, circ5912 has been shown to repress transforming growth factor β signaling, which suppresses proliferation, invasion and migration of bladder cancer induced by mesenchymal-to epithelial transition.

Conclusions: Our study firstly demonstrate that circ5912 regulates mesenchymal-to epithelial transition pathway to suppress bladder cancer progression and propose new therapeutic targets and biomarkers for bladder cancer.

Materials and Methods: Clinical values of circ5912 in human bladder cancer were examined in a cohort of 58 patients by qPCR. 2 bladder cancer cell lines, T24 and SW780, were used for biological evaluation of circ5912. CCK8, clone formation, wound healing and trans-well assays were performed to determine the *in vivo* effect of circ5912; a mouse subcutaneous model was designed for *in vivo* analysis. Western blotting, RNA pulldown assays and florescent *in situ* hybridization were applied for mechanistic analysis.

## INTRODUCTION

Bladder cancer is the 7^th^ most frequently diagnosed cancer worldwide [1]. Despite recent advances in the early diagnosis of bladder cancer, some undetectable patients progress quickly into having advanced tumors, and the reason behind the progression is not clear [1, 2]. Cancer behaviors are determined by gene expression. Hence, to further understand the underlying mechanism for cancer progression, deep molecular exploration is needed.

Circular RNAs are a group of covalently closed loop RNAs [3]. Studies have shown that circular RNAs play an important role in cancer progression [4]. According to their characteristics of being extremely stable and abundant and having cancer-specific expression profiles, the study of circular RNAs has 2 main applications [5]. On the one hand, circular RNAs act as potential biomarkers for patient diagnosis and prognosis, which is suggested by a growing number of studies. Upregulated circPRMT5 in serum and urinary exosomes [6] and downregulated circ-ITCH [7], circHIPK3 [8] and circMTO1 [9] in bladder cancer tissues are positively associated with grading, staging, infiltration, and lymph node metastasis in bladder cancer patients. In addition, circular RNAs are thought to be potential bio-targets in cancer. It was found that circular RNAs enable the regulation of cancer progression through micro RNA (miRNA) sponging [5]. For example, ciRS-7 suppresses bladder cancer growth by sponging miR-135a, which elevates P21 levels [10]; circular RNA CEP128 promotes bladder cancer cell propagation and migration via sponging mir-145-5p to regulate MAPK (mitogen-activated protein kinase) signaling [11]; and circHIPK3 decreases lung metastasis by sponging miR-558 to suppress heparanase expression [8]. Although many circular RNAs were reported to play a significant role in cancer, further work is needed to understand cancer genetics based on circular RNAs. Hence, there is an urgent need to characterize more circular RNAs and define their associated molecular mechanism in cancer.

In this study, we reanalyzed our previous RNA-seq data from 2 pairs of bladder cancer tissues [12]. Among the various circular RNAs with decreased expression in bladder cancer tissue compared to control tissue, circ5912 ranks among the top 5. It was found that circ5912 was significantly downregulated in bladder cancer tissues and negatively correlated with bladder cancer grade, stage, metastasis and better patient outcome. *In vitro* and *in vivo*, circ5912 has been shown to inhibit proliferation, invasion and migration of bladder cancer. Effective circ5912 activity enables to reverse the effect of TGF-β2 (transforming growth factor-β2)-induced EMT (epithelial-to-mesenchymal transition). In summary, our study first suggests that circ5912 suppresses bladder cancer progression by inducing the MET (mesenchymal-to epithelial transition), which suggests its potential as a biomarker for detecting bladder cancer.

## RESULTS

### circ5912 is expressed at lower levels in bladder cancer and correlates with better patient outcomes

**Figure 1:**
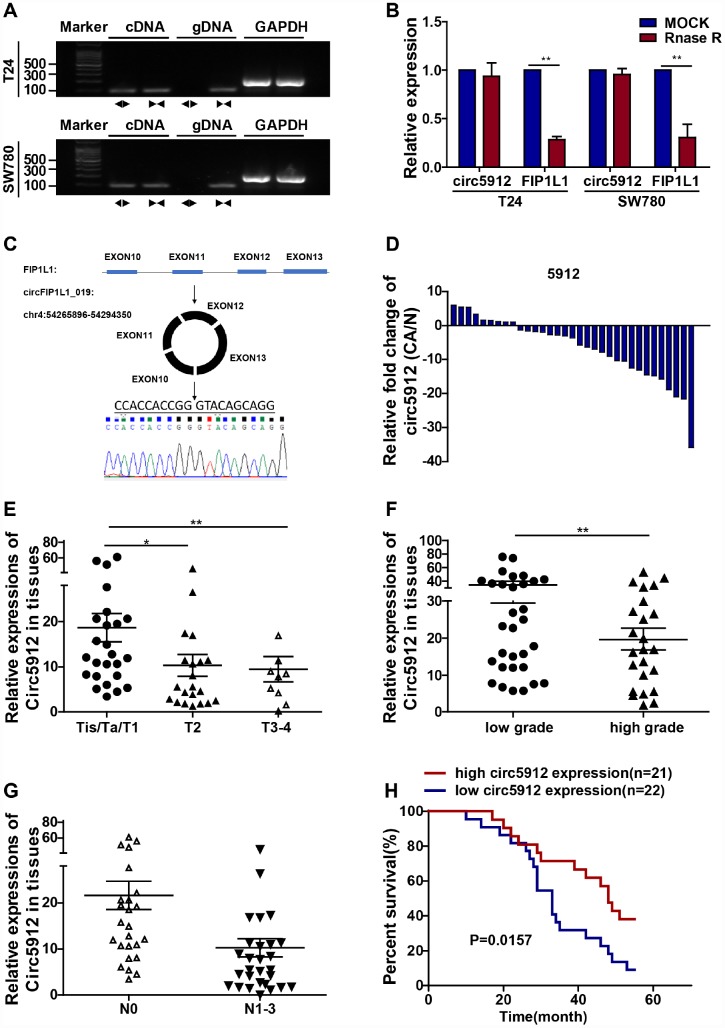
**Lower circ5912 levels are associated with advanced bladder cancer.** Divergent (circ5912, ◄►) and convergent (*FIP1L1*, ►◄) primers were designed; to prove circ5912 is circular RNA, (**A**) agarose gel electrophoresis and (**B**) RNAse treatment assays were performed; (**C**) illustration of the circulation of circ5912; (**D**) expression of circ5912 in 45 paired bladder cancer tissues; 58 bladder cancer tissues were evaluated and analyzed by: (**E**) stages, (**F**) tumor grade and (**G**) metastasis; (**H**) overall survival of 43 bladder cancer patients in following was analyzed based on the level of circ5912.

According to our previously analyzed RNA-seq data from 2 bladder cancer tissues, circ5912 ranks in the top 5 among all reduced circular RNAs in bladder cancer compared with normal control tissue. To further explore the value of circ5912, identification and characterization of circ5912 to prove that it is a circular RNA involved in cancer is of vital importance. Agarose gel electrophoresis (Figure 1A) and RNAse R treatment (Figure 1B) showed that only the genomic DNA (gDNA) of circ5912 was lost, while the cDNA of circ5912, cDNA and gDNA of *FIP1L1*, the linear form of circ5912, remained stable. In addition, sanger sequencing indicated that the sequence of circ5912 is the same as the hsa_circ_0005912 sequence found in http://circbase.org/. These results suggest that circ5912 is a circular RNA (Figure 1C). Next, we examined the clinical value of circ5912 in human bladder cancer in a cohort of 58 patients. It was observed that circ5912 expression was significantly lower in 45 paired bladder cancer tissues (Figure 1D); in 58 bladder cancer tissues, higher circ5912 levels associates with better clinical pathological conditions, including stage (Figure 1E), tumor grade (Figure 1F) and metastasis (Figure 1G); additionally, a longer overall survival time was observed among 43 patients for whom circ5912 was regarded as high (Figure 1H). Taken together, these results suggest that circ5912 is expressed at lower levels in bladder cancer and that a higher level of circ5912 correlates with better patient outcomes. The general patient information is listed in Table 1.

**Table 1:** Relationship between circ5912 level and clinical characteristics in bladder cancer.

**Total**	**Patients**	**Expression of circ5912**		
		**High**	**Low**	**p values**
**Age(mean)**	53.21	52.38	54.12	0.535
**Gender**				
Male	46	20	26	0.647
Female	12	6	6	
**Tumor stage**				
Tis/Ta/T1	28	18	10	<0.01
T2	21	6	15	
T3/T4	9	2	7	
**Grade**				
High	27	20	7	0.008
Low	31	6	25	
**Number of tumors**				
Solitary	41	20	21	0.819
Multiple	17	6	11	
**Lymph node metastasis**				
Negative	26	16	10	0.016
Positive	32	10	22	
**Follow-up (month, mean)**	33.23	32.73	35.08	0.0157

### Silencing circ5912 in bladder cancer cells promotes cell growth and metastasis *in vitro*

**Figure 2:**
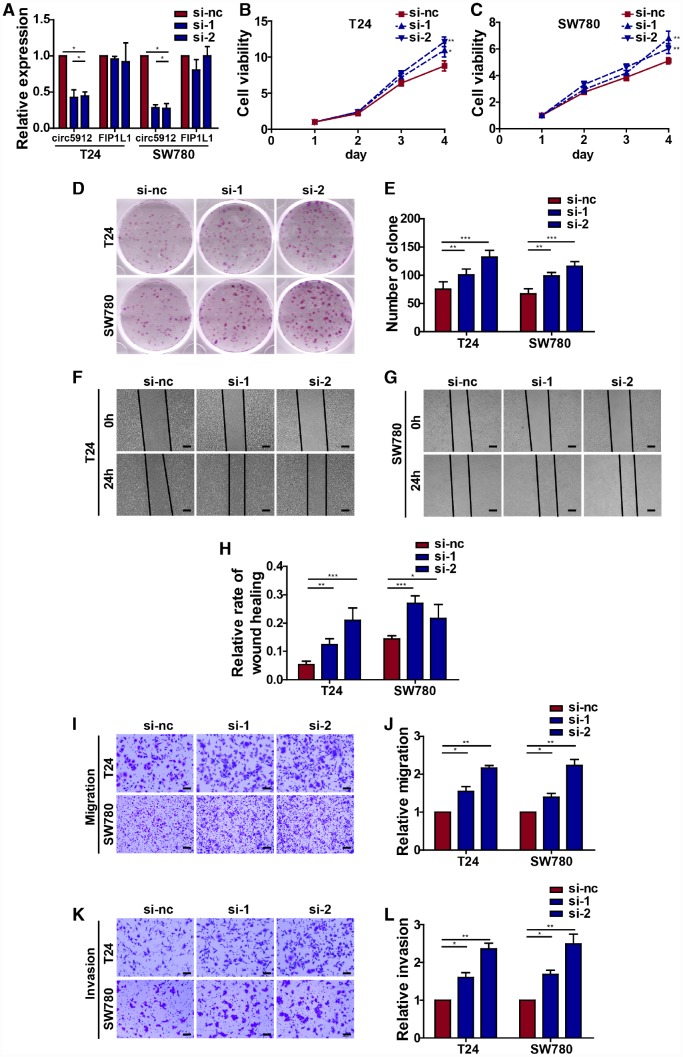
**Silencing circ5912 promotes bladder cancer cell growth and metastasis *in vitro****.* Two siRNAs that targeted circ5912 were designed and synthesized. (**A**) qPCR detected levels of circ5912 and *FIP1L1* after treatment with the siRNAs; (**B**, **C**) a CCK8 assay was performed to evaluate cell viability; (**D**, **E**) a clone forming assay was performed to detect the ability of self-renewal; (**F**–**H**) wound healing ability was measured by the distance between the two sides of induced injury after 24 hours, scale bar: 100μm; (**I**–**L**) migration and invasion were assessed by counting cells that were able to penetrate the trans-well membrane, scale bar: 25μm.

As we mentioned before, circ5912 suggests a tumor suppressive role in bladder cancer with regard to clinical parameters. To subsequently prove a tumor suppressive role of circ5912 in bladder cancer, we first designed 2 siRNAs to silence circ5912 in 2 bladder cancer cell lines, T24 and SW780. After treatment of bladder cancer cells with the siRNAs, the expression of circ5912 decreased 4.3-fold in T24 cells and 3.6-fold in SW780 cells, while the level of *FIP1L1* remained unchanged (Figure 2A). Next, a CCK8 assay was performed to evaluate the cell viability of bladder cancer cells after treatment with siRNAs. It was found that silencing circ5912 apparently promoted viability of bladder cancer cells (Figure 2B, 2C), and an improved clone-forming ability was observed (Figure 2D, 2E). These results suggest that silencing circ5912 promotes bladder cancer growth. In addition, to detect the effect of circ5912 on the migratory capacity of bladder cancer cells, wound healing and trans-well assays were performed. It was found that silencing circ5912 encouraged the wound healing capability of bladder cancer (Figure 2F–2H), as well as the migration and invasion potential (Figure 2I–2L). Taken together, the above results suggest that silencing circ5912 promotes bladder cancer cell growth and migratory capacity *in vitro*.

### Overexpression of circ5912 suppresses bladder cancer growth and metastasis

**Figure 3:**
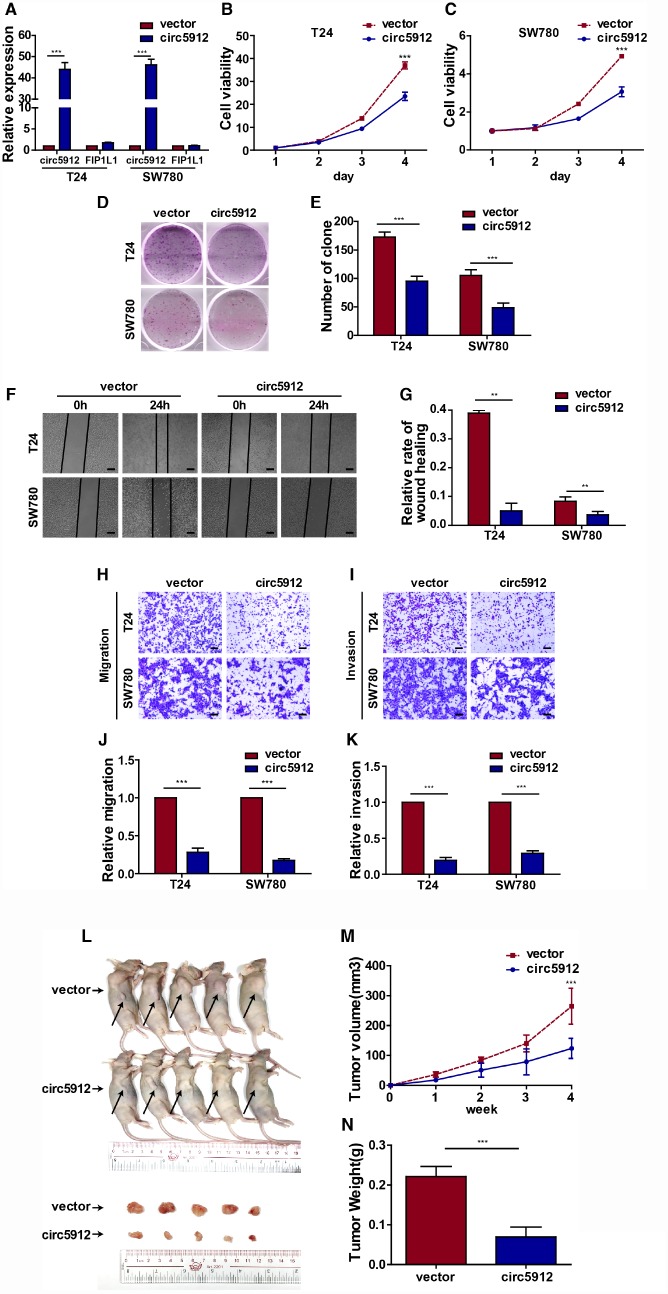
**Overexpression of circ5912 suppresses bladder cancer growth and metastasis.** Bladder cancer cell lines with overexpressed circ5912 were designed and produced. (**A**) qPCR detected levels of circ5912 and *FIP1L1* after circ5912 overexpression; (**B**, **C**) a CCK8 assay was performed to evaluate cell viability; (**D**, **E**) clone-forming ability was detected; (**F**, **G**) wound healing ability was measured by the distance between the two sides of induced injury after 24 hours, scale bar: 100μm; (**H**–**K**) migration and invasion were assessed by counting cells that penetrated the trans-well membrane, scale bar: 25μm; (**L**–**N**) the *in vivo* effect of circ5912 was evaluated by subcutaneously injecting circ5912 overexpressing cells into nude mice. Mice were killed 4 weeks after injection, and tumor weight and volume were measured.

We then constructed the circ5912-overexpressing bladder cancer cell lines in T24 and SW780 cells. The overexpression of circ5912 had less effect on *FIP1L1* expression (Figure 3A). In contrast to silencing, overexpression significantly weakened bladder cancer cell viability (Figure 3B, 3C) as well as clone formation (Figure 3D, 3E). Cell growth strongly relies on the balance between proliferation and apoptosis. We next performed an Annexin V/Pi apoptotic assay to prove that the above reduction in cell viability did not correlate with apoptosis (Supplementary Figure 1A, 1B). As we thought, there were no significant differences in the apoptotic phenotype after circ5912 overexpression. These results suggest that overexpression of circ5912 suppresses bladder cancer growth. Next, wound healing and trans-well assays were applied to evaluate the effect of circ5912 overexpression on the migratory capacity of bladder cancer cells. It was found that overexpressed circ5912 decreased the wound healing capability of bladder cancer (Figure 3F, 3G), as well as the migration (Figure 3H, 3J) and invasion (Figure 3I, 3K) potential. The above results suggest that overexpression of circ5912 in bladder cancer cells suppresses cell growth, migration and invasion *in vitro*. To explore the *in vivo* effect of circ5912, a mouse subcutaneous tumor model was used. Injection of circ5912-overexpressing cells into nude mice formed tumors with slower growth and lighter weight than tumors formed by normal cell injection (Figure 3L, 3M, 3N). Taken together, the above results suggest that overexpression of circ5912 suppresses bladder cancer growth and metastasis.

### circ5912 reverses TGF-β2-induced EMT in bladder cancer

**Figure 4:**
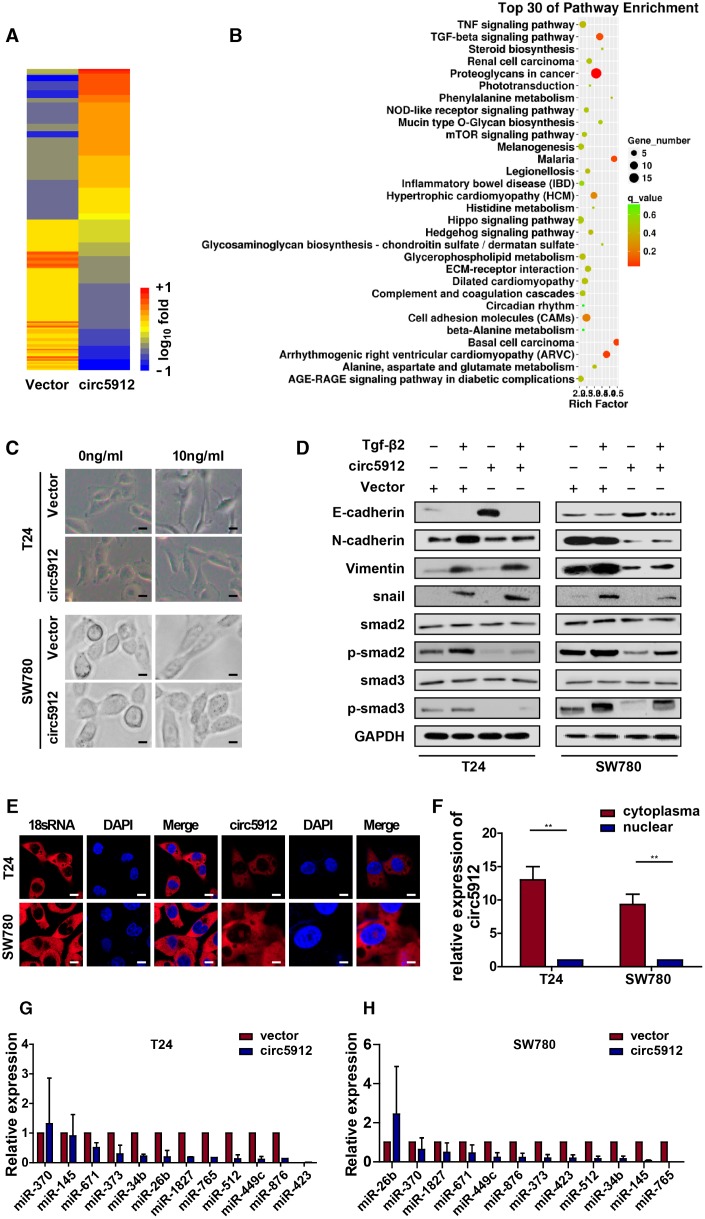
**circ5912 reverses Tgf-β2-induced EMT in bladder cancer.** To identify the underlying mechanism of circ5912, (**A**, **B**) The mRNA sequence was analyzed for the downstream alteration after circ5912 overexpression; cells were treated with TGF-β2 in 10ng/ml for 24 hours to induce EMT. (**C**) the bladder cancer phenotype was observed under light microscope, scale bar: 2.5μm; (**D**) western blot analysis was performed to determine the levels of different endothelial and mesenchymal markers; (**E**) FISH and (**F**) nuclear-plasma extraction assays were performed to detect the location of circ5912, scale bar: 2.5μm. Cytoplasmic 18s RNA was used as a positive control; (**G**, **H**) RNA pulldown assay showed the binding properties of multiple miRNAs and circ5912.

We have proven that circ5912 suppresses bladder cancer growth and metastasis, but the underlying mechanisms remain less well understood. Therefore, we performed mRNA sequence analysis after circ5912 was overexpressed. Among 348 altered genes, Vimentin and Tgf-β2 were significantly reduced (Figure 4A), as well as genes involved in TGF-β signaling pathways which are the main mediators of cancer EMT (Figure 4B). Besides, expression of snail, slug, twist, zeb2 were coordinately repressed (Figure 4A); activation of these transcriptional factors was characterized as EMT processing. Hence, the results suggest that circ5912 may participate in MET process to suppress bladder cancer progression. To prove our hypothesis, recombinant TGF-β2 treatment was used to induce EMT in bladder cancer cells. Cells displayed spindle-like structures after treatment with TGF-β2 compared with sharp edges exhibited by control cells (Figure 4C); at the same time, the expression of E-cadherin, an epithelial marker, was downregulated, whereas the expression of N-cadherin, Vimentin and snail, which are mesenchymal markers [11, 12], was increased (Figure 4D). These results suggest that TGF-β2 successfully induced EMT in bladder cancer cells. Next, to prove the role of circ5912 in regulating MET, we compared the cell morphology and EMT marker expression among circ5912-overexpressing cells and normal control cells after treatment with TGF-β2. It was found that circ5912 obviously inhibited EMT induction of TGF-β2, as demonstrated by a slight spindle-like structure (Figure 4C), higher E-cadherin, and lower N-cadherin, Vimentin and snail expression (Figure 4D), compared with the normal control. Besides, the expression of E-cadherin, N-cadherin and Vimentin were also determined by immune-histochemistry in tissues from our xenograft models. As expected, expression of E-cadherin increased definitely, while level of N-cadherin and Vimentin decreased when circ5912 was over-expressed. The results have been provided in (Supplementary Figure 2). Taken together, these results suggest that circ5912 participates in the MET process in bladder cancer cells. The molecular role strongly relies on the cellular location. Cytoplasmic circular RNA may likely play a posttranscriptional role, such as sponging of miRNA. To further explore the mechanisms of circ5912 in bladder cancer, florescent *in situ* hybridization (Figure 4E) and nuclear-cytoplasm extraction assays (Figure 4F) were performed, and circ5912 was predominantly observed in the cytoplasm of bladder cancer cells. Subsequently, 12 miRNAs that were predicted by Targetscan and miRanda to bind circ5912 were analyzed. An RNA pulldown assay was performed, and there were no significant results for circ5912 binding in either of the bladder cancer cell types (Figure 4G, 4H). These results suggest that circ5912 may exert its role through another posttranscriptional mechanism, not merely miRNA sponging.

## DISCUSSION

Through high-throughput RNA sequencing and bioinformatics analysis, multiple circular RNAs have recently been identified as extremely stable, abundant RNAs with cell-type specific expression in cancer [4, 13, 14]. Effective circular RNA activities, on the one hand, serve as potential biomarkers for detection in cancer patients; on the other hand, they play an important role in cancer progression. For example, circ-ITCH [7], circHIPK3 [8] and circMTO1 [9] were all identified to have reduced levels in bladder cancer tissues and were negatively correlated with grading, staging, invasion, and lymph node metastasis of bladder cancer; circPRMT5 was significantly upregulated in serum and urine exosomes of bladder cancer patients and was statistically associated with tumor metastasis [6]. In addition, ciRS-7 has been reported to inhibit cancer growth by upregulating P21 [10], circELP3 promotes bladder cancer progression and drug resistance [12], circHIPK3 reduces lung metastasis by inhibiting heparanase expression [8], and circular RNA CEP128 promotes bladder cancer cell migration via MAPK signaling [11]. Taken together, these data suggest that circular RNA serve as a promising target in cancer research.

In the present study, we found that circ5912 was significantly downregulated in bladder cancer tissues and inversely associated with grade, stage, metastasis, and longer overall survival in bladder cancer patients. These results suggest that circ5912 can be a potential biomarker for the diagnosis and follow-up of bladder cancer, as many people are reluctant to receive invasive cystoscopy [2]. Therefore, future studies are expected to have a larger sample cohort, not only from tissues but also from patients' serum or urine, to further analyze the potential of circ5912 as a biomarker for bladder cancer. Moreover, we have also demonstrated that circRIP2 suppresses the proliferation, invasion and migration of bladder cancer *in vitro* and *in vivo*. To further explore the underlying mechanisms of circ5912, the mRNA sequence was analyzed, and it was found that TGF-β signaling pathways were changed significantly. TGF-β signaling coordinates a complex signaling network to regulate tumorigenesis and cancer progression through EMT [15, 16, 17]. Moreover, compared with normal controls, overexpression of circ5912 significantly deprived TGF-β2-induced EMT, with a lighter spindle-like structure, higher E-cadherin and lower snail, N-cadherin and Vimentin expression. These results indicate that circ5912 may be involved in the MET process to inhibit bladder cancer progression. EMT plays an ultimate role in cancer progression [15]. In addition to driving cancer metastasis and growth, EMT has been identified to play a role in maintaining cancer pluripotency [18] and drive cancer immunity [19, 20, 21]. These findings suggest that circ5912 may work more broadly than its currently understood role in cancer growth and metastasis. Hence, further studies are encouraged to evaluate the role of circ5912 with regard to the devastating background of EMT in cancer. In addition, during the anchorage of circulating metastatic cancer cells, effective MET enables cells to more easily localize and survive in the secondary metastatic site [22]. Hence, whether circ5912 may work separately during the process of cancer progression. For example, circ5912 suppresses cancer progression in the early phage but promotes cancer development if distant metastasis is formed. However, this hypothesis needs further study.

## CONCLUSIONS

Our study first investigates the role of circ5912 in bladder cancer, not only as a potential diagnostic biomarker but also as a specific inhibitor of the growth of bladder cancer. Further, this study is also the first to explore how circular RNA is involved in the MET process to inhibit bladder cancer progression. We hope that these findings will provide new perspectives for a better understanding of bladder cancer biology; a larger follow-up study will help to gain a deeper understanding of the potential role of circ5912 as a biomarker for the diagnosis of bladder cancer.

## MATERIALS AND METHODS

### Human tissue management

58 bladder cancer tissues, 45 paired bladder cancer tissues and adjacent normal controls were collected from 1^st^ June 2014 to 1^st^ March 2019 at Sun Yat-Sen Memorial Hospital: Only 43 patients were followed finally. All studies involved in tissue detection were permitted by the Ethics Committee of Sun Yat-Sen University. Permission to use tissues experimentally was granted by all patients involved. All pathologic and histological diagnosis were confirmed by 3 pathologists.

### Cell culture

Bladder cancer cell lines T24 and SW780 were purchased from ATCC and cultured in RPM-1640 medium that contained 100 U/ml penicillin and streptomycin (Gibco, USA) and 10% FBS (Gibco, USA). Cells were cultured under humidified, constant 37°C and 5% CO2 atmosphere. The T24 cell line was tested by the short tandem repeat method on 20^th^ Nov. 2018, while SW780 was tested on 18^th^ July 2018.

### RNA preparation and quantitative real-time PCR

RNA was extracted by a rapid RNA extraction kit (ES Science, China) and reverse transcribed by the PrimerScriptTM RT Master Mix Kit (TakaRa, Japan). Quantitative real-time PCR (qPCR) was performed to detect RNA levels with a TB Green Premix Ex Taq II Kit (TakaRa, Japan) with a QuantStudioTM DX Real-Time PCR instrument (Applied Biosystems, Thermo, USA). The primers used in this study are listed below: circ5912-F: AGCAAACCACCTCCGTTTTTC; R: TGGAAGGGCAGTTTCTTTCTC; FIP1L1-F: AATGC CAGTGCTAATCCTCCA; R: TCATCTTCGGTCTCA GTCACTT; GAPDH-F: TACTAGCGGTTTTACG GGCG; R: TCGAACAGGAGGAGCAGAGAGCGA.

### Agarose gel electrophoresis and RNAse R treatment

Detailed procedures are explained in our previous research [14].

### Fluorescent *in situ* hybridization (FISH)

The FISH probe for circ5912 was synthesized by Genepharm (Suzhou, China). The FISH assay was performed according to procedures recommended by RiboBio (Guangzhou, China). 18S RNA was used as a positive control. (sequence of circ5912 FISH probe was: cy3-CTGCCTCCAGAAAAGCCAATTCAAGCGTTGGGAAGTGGCAGGATCGATAT-cy3) Cells were observed under a laser scanning confocal microscope (LSM 800 with Airyscan, ZEISS, German).

### Nuclear and cytoplasmic extraction assay

A Thermo Fisher Kit (78833, German) was used to extract nuclear and cytoplasmic RNA.

### Cell transfection

siRNAs targeting circRIP2 were purchased from Genepharm (Suzhou, China). Lipofectamine iMAX (Gibco, USA) was used for transfection. Sequence of siRNAs for circ5912 are listed below (5′ -3′): circ5912 si-1 sense: GAUUCCACCACCGGGUACATT; antisense: UGUACCCGGUGGUGGAAUCTT; circ5912 si-2 sense: CCACCGGGUACAGCAGGG ATT; antisense: UCCCUGCUGUACCCGGUGGTT.

To construct stable circ5912-overexpressing bladder cancer cells, lenti-virus containing a plenty-ciR-GFP-T2A-puro vector was synthesized by Genechem (Shanghai, China), and cells were infected experimentally.

### CCK8 viability assay

A total of 1000 cells/well were plated in a 96-well plate 24 hours before counting. One hundred microliters of media that contained 10% CCK8 (Biyuntian, China) was added to each well and incubated for 2 hours, and then a microplate reader (TECAN Spark 10M, Switzerland) measured the OD values under 452 nm.

### Clone formation assay

A total of 1000 cells/well were seeded in 6-well plates. Cell clones were harvested when more than 50 cells/clone were counted. Crystal violet (1%) was used to stain the clones.

### Transwell migration and Matrigel invasion assay

A single cell suspension with 80,000 cells was inoculated into the upper chamber and mixed with 200 μl of serum-free medium for migration and 200 μl of matrigel for invasion. A total of 600 μl of medium containing 10% FBS was added into the lower chamber. After 8 hours for T24 cells and 48 hours for SW780 cells, the upper chamber was harvested and fixed with 4% paraformaldehyde for 15 minutes. Subsequently, the upper chamber was stained with 0.1% crystal violet, and cells that penetrated were counted. The images were taken under a microscope.

### Wound healing assay

Cells were seeded at 100% density in 6-well plates and scraped with a 200 μl pipette tip. Scratched lines were taken at 0 hour and 24 hours. Wound healing ability was measured by counting the distance between the two sides of the scratch. The distance at 0 hour was normalized as a control. Cells were observed under a light microscope.

### Tumor subcutaneous mouse model

All animal studies were permitted by The Animal Management Committee of Sun Yat-Sen University. A total of 10^7^ circ5912 overexpressed bladder cancer cells were subcutaneously injected into nude mice (all mice were male, 3-4 weeks old). Mice were sacrificed when tumor volume (V) reached over 200 mm^3^, V=(L×W×W)/2, weight (W) and length (L).

### Western blot analysis

E-cadherin, N-cadherin, Vimentin, snail, and GAPDH antibodies were purchased from Santa Cruz (USA). The protein bands were visualized by ECL assay and exposed in a dark room.

### RNA pulldown

We have described the detailed procedures for RNA pulldown in our previous research. The specific biotin-labeled probe for circ5912 was designed and synthesized by Genepharm (Suzhou, China). The sequence of the circ5912 probe was as follows: 5′-CCAGUUCUUCCC UGCUGUACCCGGUGGUGGAAUCAGAGGU-3′.

### Statistical analysis

Statistical analysis in this article was performed by GraphPad 5.0. A t test was performed between 2 independent groups, one-way ANOVA test was applied between each group, and Kaplan-Meier curves and log rank tests were used to analyze patient survival. P < 0.05 was considered to be statistically significant.

### Ethics approval

Use of human tissues was approved by the ethics committee of Sun Yat-Sen University. All animal studies were permitted by The Animal Management Committee of Sun Yat-Sen University. All patients involved signed the contract for using their tissues experimentally.

## Supplementary Material

Supplementary Figures
