# Correction for: circ5912 suppresses cancer progression via inducing MET in bladder cancer

**DOI:** 10.18632/aging.102886

**Published:** 2020-02-27

**Authors:** Yinjie Su, Zehu Du, Guanglei Zhong, Yiyao Ya, B Junming, Juanyi Shi, Luping Chen, Wen Dong, Tianxin Lin

**Affiliations:** 1 The Department of Urology, Sun Yat-Sen Memorial Hospital, Sun Yat-Sen University, Guangzhou, China; 2 The Department of Thyroid Surgery, Sun Yat-Sen Memorial Hospital, Sun Yat-Sen University, Guangzhou, China; 3 The Department of Gynecological Oncology, Sun Yat-Sen Memorial Hospital, Sun Yat-Sen University, Guangzhou, China; 4 The Department of Urology, Guangzhou First People's Hospital, School of Medicine, South China University of Technology, Guangzhou, China; 5 The Department of Pediatric Surgery, Sun Yat-Sen Memorial Hospital, Sun Yat-Sen University, Guangzhou, China

**Keywords:** correction

Original article: Aging. 2019; 11:10826–10838.  . https://doi.org/10.18632/aging.102464

**This article has been corrected:** The authors requested the replacement of Figure 1D, E, F, G, Figure 2I, Figure 3H and Table 1. The authors made changes to improve presentation of the panels. They removed duplications and overlaps of the images with minor modifications of the original data. The revised table reflects the updated properties of the patients sample pool.

These corrections do not change the content of the publication and do not affect the conclusion of this research. The authors apologize for the unintentional mistakes.

The corrected Figures and Table are provided below.

**Figure 1:**
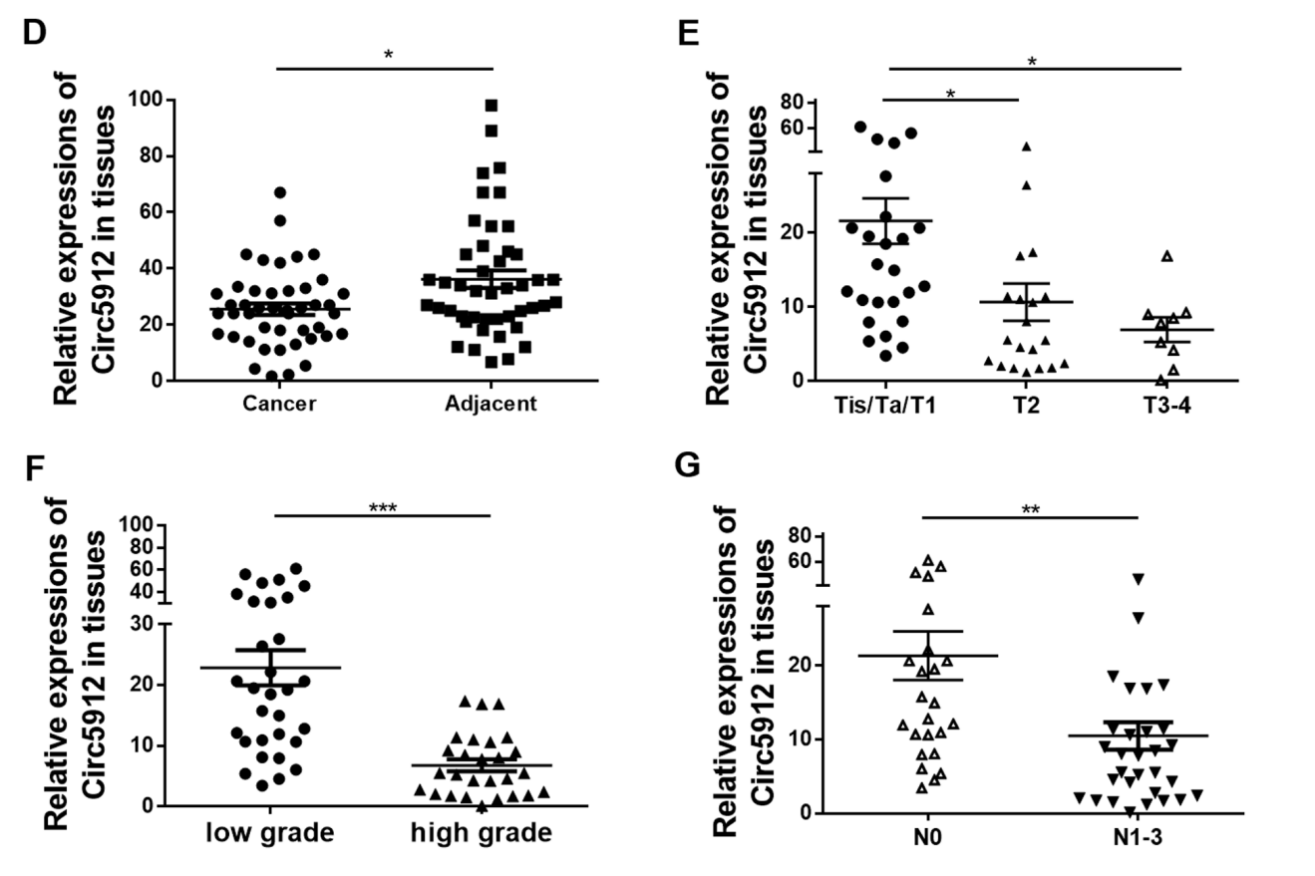
**Lower circ5912 levels are associated with advanced bladder cancer. **(**D**) Expression of circ5912 in 45 paired bladder cancer tissues; 58 bladder cancer tissues were evaluated and analyzed by: (**E**) stages, (**F**) tumor grade and (**G**) metastasis; (**H**) overall survival of 43 bladder cancer patients in following was analyzed based on the level of circ5912.

**Figure 2:**
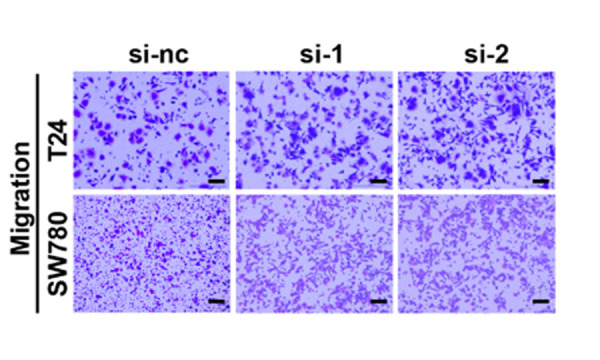
**Silencing circ5912 promotes bladder cancer cell growth and metastasis *in vitro***. (**I**) migration and invasion were assessed by counting cells that were able to penetrate the trans-well membrane, scale bar: 25μm.

**Figure 3:**
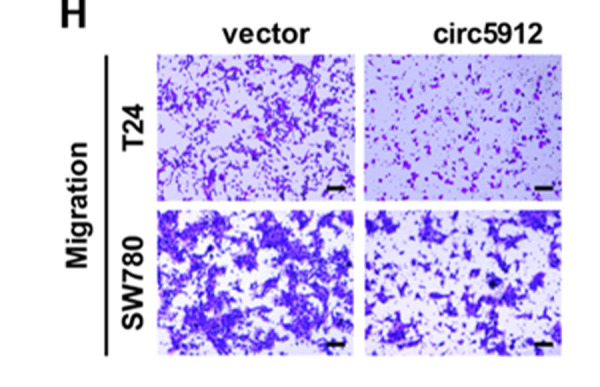
**Overexpression of circ5912 suppresses bladder cancer growth and metastasis.** (**H**) migration and invasion were assessed by counting cells that penetrated the trans-well membrane, scale bar: 25μm.

**Table 1:** Relationship between circ5912 level and clinical characteristics in bladder cancer.

**Total**		**Patients**	**Expression of cic5912**		
			**High**	**Low**	**p**
**Age(mean)**		56	52.52	59.48	0.200
**Gender**					
	Male	46	20	26	0.052
	Female	12	9	3	
**Tumor stage**					
	Tis/Ta/T1	28	20	8	0.003
	T2	21	8	13	
	T3/T4	9	1	8	
**Grade**					
	High	27	6	21	<0.001
	Low	31	23	8	
**Number of tumors**					
	Solitary	41	21	20	0.773
	Multiple	17	8	9	
**Lymph node metastasis**					
	Negative	26	18	8	0.008
	Positive	32	11	21	
**Follow-up (month, mean)**		38.465	43.095	34.045	0.016

