# Supplementary material for: circ5912 suppresses cancer progression via inducing MET in bladder cancer
Source: Aging (Albany NY). 2019 Dec 5;11(23):10826–38. doi: 10.18632/aging.102464 (PMC6932894; doi:10.18632/aging.102464)
Supplement: Supplementary Figures [file aging-11-102464-s001..pdf]

SUPPLEMENTARY FIGURES

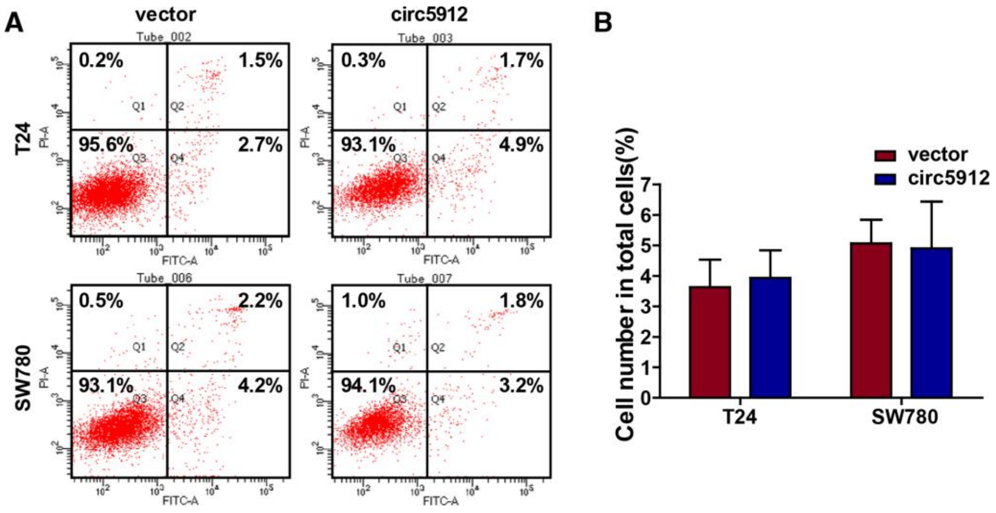

Supplementary Figure 1. (**A**, **B**) An annexin V/Pi apoptotic assay showed an effect of circ5912 on bladder cancer cell apoptosis.

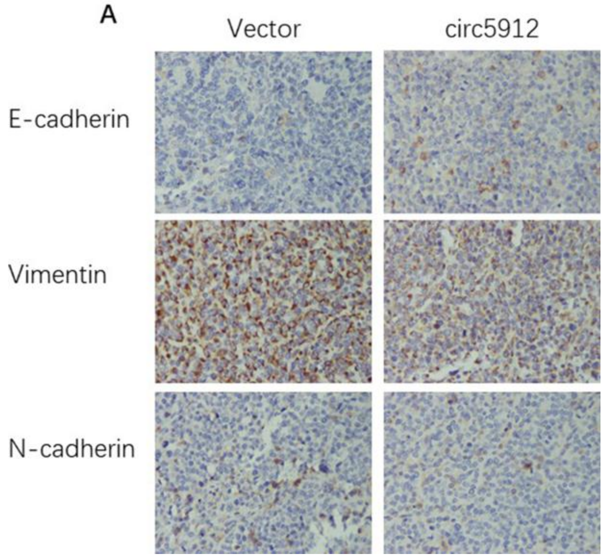

Supplementary Figure 2. (**A**) The expression of E-cadherin, N-cadherin and Vimentin were determined by immune-histochemistry in tissues from our xenograft models. Photos were captured under 400× light microscope.
